# Oxidative Stress in Mouse Sperm Impairs Embryo Development, Fetal Growth and Alters Adiposity and Glucose Regulation in Female Offspring

**DOI:** 10.1371/journal.pone.0100832

**Published:** 2014-07-09

**Authors:** Michelle Lane, Nicole O. McPherson, Tod Fullston, Marni Spillane, Lauren Sandeman, Wan Xian Kang, Deirdre L. Zander-Fox

**Affiliations:** 1 Discipline of Obstetrics and Gynaecology, School of Paediatrics and Reproductive Health and Robinson Institute, University of Adelaide, South Australia, Australia; 2 Repromed, Dulwich, South Australia, Australia; Louisiana State University and A & M College, United States of America

## Abstract

Paternal health cues are able to program the health of the next generation however the mechanism for this transmission is unknown. Reactive oxygen species (ROS) are increased in many paternal pathologies, some of which program offspring health, and are known to induce DNA damage and alter the methylation pattern of chromatin. We therefore investigated whether a chemically induced increase of ROS in sperm impairs embryo, pregnancy and offspring health. Mouse sperm was exposed to 1500 µM of hydrogen peroxide (H_2_O_2_), which induced oxidative damage, however did not affect sperm motility or the ability to bind and fertilize an oocyte. Sperm treated with H_2_O_2_ delayed on-time development of subsequent embryos, decreased the ratio of inner cell mass cells (ICM) in the resulting blastocyst and reduced implantation rates. Crown-rump length at day 18 of gestation was also reduced in offspring produced by H_2_O_2_ treated sperm. Female offspring from H_2_O_2_ treated sperm were smaller, became glucose intolerant and accumulated increased levels of adipose tissue compared to control female offspring. Interestingly male offspring phenotype was less severe with increases in fat depots only seen at 4 weeks of age, which was restored to that of control offspring later in life, demonstrating sex-specific impacts on offspring. This study implicates elevated sperm ROS concentrations, which are common to many paternal health pathologies, as a mediator of programming offspring for metabolic syndrome and obesity.

## Introduction

There is increasing recognition that paternal health at conception can impact on the health of subsequent offspring. For example human studies have linked paternal smoking to an increase in the occurrence of childhood cancer [1], [2], likely due to the direct impact of mutagenic xenobiotics found in cigarette smoke increasing the mutation load in sperm DNA [3]. Advanced paternal age has been linked to a higher incidence of childhood autism spectrum disorders [4], most likely due to an increase in single nucleotide polymorphisms and copy number variations in sperm [5]. Occupational solvent exposure in men increases congenital malformations in fetus [6] and in addition paternal obesity reduces pregnancy establishment [7]–[9] increases childhood BMI [10] and susceptibility to disease later in life [11]–[15]. However, although some changes in the epigenetic makeup of sperm have been reported, the programming mechanism which transmits the altered paternal health cues from the sperm to the embryo, pregnancy and offspring is currently unknown.

Reactive oxygen species (ROS) are normal by-products of metabolism, which, at physiological levels, play important roles as regulatory mediators in signalling processes important for sperm capacitation and the acrosome reaction [16]. In healthy cells, or cells not undergoing oxidative stress, the concentration of ROS is controlled by antioxidants, however when unregulated, elevated concentrations can induce DNA damage and alter methylation patterns in sperm [17]–[20]. Sperm are particularly susceptible to oxidative damage due to their lack of cytoplasm which is mostly removed during the final stages of spermiogenesis [21], [22]. This ultimately renders sperm vulnerable to oxidative damage due to diminished amounts of defensive enzymes, such as catalase and glutathione peroxidase, which are involved in protecting cells from ROS associated damage [23].

Interestingly a wide range of paternal diseases and environmental exposures including cancer, smoking, obesity, chemical exposure and ageing all elevate the concentration of reactive oxygen species (ROS) in sperm [24], [25]. Studies in both humans and rodent models have established that increased ROS levels are associated with increased DNA damage in sperm, which in turn is associated with poorer pregnancy outcomes [17]–[19]. However these studies are only associative and a direct correlation between increased sperm ROS concentrations and decreased embryo viability has not been made. The direct effect of increased ROS in sperm, independent of upstream mediators, on subsequent embryo development and pregnancy has yet to be explored. Whether this may be a convergence point for the impact of multiple distinct paternal pathologies on pregnancy and offspring health is unknown. Therefore we investigated the direct effect that increased sperm intracellular ROS by exposure to H_2_O_2_ has on sperm function and subsequent embryo, pregnancy and offspring health.

## Materials and Methods

### Ethics Statement

The animal ethics committee of the University of Adelaide approved all experiments, and animals were handled in accordance with the Australian Code of Practice for the Care and Use of Animals for Scientific Purposes. All mice were maintained at 24°C on a 12:12 h dark/light cycle with food and water provided ad libitum. Embryo transfer surgery and DEXA was performed under 2% Avertin (2, 2, 2-Tribromoethanol, 2-methyl-2-butanol) anesthesia, and all efforts were made to minimize suffering. Where required, analgesic of 0.1 mg/kg body weight of Temegesic (Buprenorphine) was administered by intraperitaneal injection after surgery.

### Generation of increased ROS in sperm

Sperm was collected from 30 C57Bl6 × CBA (CBAF1) male mice (8–10 weeks of age) from vas deferens and the caudal epididymis. Sperm were collected in 1 ml of pre-equilibrated medium (G-IVF, Vitrolife, Goteborg, Sweden) at 37°C and allowed to swim out for 10 mins. After 10 mins sperm from individual males were split (500 µl each) into either control G-IVF medium or G-IVF medium supplemented with 1500 µM H_2_O_2_ (this concentration increases ROS levels to a similar level as that seen in an obese male mouse, data not shown) for 1 h, such that both treatments used sperm from the same individual animal. After 1 h of incubation sperm samples were assessed for motility, intracellular ROS levels, sperm binding and then used for in vitro fertilisation.

### Sperm motility and morphology

Sperm concentration and motility was assessed in accordance to World Health Organization (WHO) 2010 manual for the process of human semen for medical purposes [26].

### Sperm intracellular and mitochondrial generation of ROS

Intracellular ROS levels were assessed in progressively motile sperm by incubation with 1 µM DCFDA (2′,7′-dichlorodihydrofluorescein diacetate, Invitrogen) for 20 min at 37°C [27]. Sperm were then washed twice and imaged individually using a fluorescent microscope with photometer attachment. For normalisation of dye uptake and cleavage, sperm samples were normalised to 5-(and 6)-carboxy-2′,7′-dichlorofluorescein diacetate as a measure of intracellular esterase activity (carboxy-DCFDA; Invitrogen).

For assessment of mitochondrial ROS, sperm were incubated in 2 µM MitoSox Red (MSR, Molecular Probes, Eugene) for 301 min in 6% CO_2_-5% O_2_ at 37°Cas previously described [28]. MSR fluorescence was measured on a FACSCanto flow cytometer (BD Bioscience, North Ryde, Australia).Non-specific sperm events were gated out and 50,000 cells were examined per sample. MSR results were expressed as the percentage of sperm positive for MSR.

### Assessment of lipid peroxidation in sperm

The amount of sperm lipid peroxidation in sperm was assessed by measuring 4-hydroxy-2-nonenal (4-HNE) levels. Sperm were fixed in 4% paraformaldehyde followed by permeabilisation in 0.5% Triton X-100 for 45 min and incubation in 10% donkey serum for 30 min at room temperature. After a 4°C overnight incubation in 4-HNE antibody (1∶100; Jomar Bioscience) sperm were incubated in secondary antibody (Alexa fluor 488 donkey anti-rabbit IgG, Life Technologies) at 1∶200 dilution for 2.5 h at room temperature. 4-HNE fluorescence was measured on a FACSCanto flow cytometer (BD Bioscience, North Ryde, Australia). Again, non-specific sperm events were gated out and 20,000 cells were examined per sample. Results were expressed as the percentage of sperm positive for 4-HNE.

### Oocyte collection and sperm binding

Sperm binding was performed as previously described [27]. Briefly Swiss female mice (3–4 week old) were superovulated by intraperitaneal (IP) injection of 5 IU of pregnant mare's gonadotrophin (PMSG; Folligon; Intervet, Bendigo, Victoria, Australia). This was followed 46–48 h later by an IP injection of 5 IU human chorionic gonadotrophin (hCG; Pregnyl; Organon, Oss, The Netherlands) as previously described. COCs were collected 12 h after hCG injection into G-IVF at 37°C, 6% CO_2_, 5% O_2_ and 89% N_2_. COCs were inseminated with 1×10^4^ motile sperm/mL. For assessment of sperm binding to the zona pellucida, oocytes were co-incubated with sperm for 1 h and the number of sperm bound to the zona pellucida counted. For generation of embryos, gametes were co-incubated for 4 h.

### Embryo culture and development

Putative zygotes were washed in G1 culture medium (Vitrolife) and cultured at 37°C, 6% CO_2_, 5% O_2_ and 89% N_2_. Fertilization was indicated by cleavage to the 2-cell stage after 24 h of culture. On-time embryo assessments were determined for 8-cell development after 48 h of culture, morula and early blastocyst development after 80 h of culture and blastocyst expansion and hatching 96 h after culture with all development being expressed as a proportion of 2-cell cleavage.

### Blastocyst cell number and differentiation

Allocation of cells to the trophectoderm (TE) and inner cell mass (ICM) in the blastocyst, was performed as previously described [29]. Briefly, blastocysts were placed into 0.5% pronase (Sigma Chemical Company, St Louis, MO, U.S.A) at 37°C until the zona pellucida dissolved, before incubation in 10 µM of 2,4,6-trinitrobenzenesulfonic acid (TNBS, Sigma) at 4°C for 10 min. Blastocysts were transferred into 0.1 mg/µl anti-dinitrophenyl- BSA (anti-DNP, Sigma) for 10 min at 37°C and then placed in guinea pig serum (Sigma) with PI (Sigma) for 5 min at 37°C. Finally, embryos were placed in bisbenzimide (Sigma) at 4°C overnight. The following day the embryos were washed through 100% ethanol, mounted in glycerol and visualized using a fluorescence microscope.

### Embryo transfer

Embryo transfer experiments were conducted to examine embryo viability using 8–10 week-old 3.5 day old pseudo-pregnant female Swiss mice (i.e. previously mated with vasectomised Swiss male mice). To ensure synchronicity into pseudo pregnant females, morphological normal blastocysts (expanded and hatching blastocysts) cultured for 96 h were vitrified in pools of 6 using RapidVitBlast and the Rapid-I vitrification straw (Vitrolife) as per manufacture instructions. The number of embryos warmed was determined based on the number of pseudo pregnant females for transfer. Warming was performed on the morning of embryo transfer using Vitrolife Warm kit, with post thawed embryos pooled per treatment and placed into GMOPS (Vitrolife) for 30 min prior to transfer. Warming survival was 90–95% for both groups. For assessment of day 18 implantation and fetal development, six morphological normal expanded or hatching blastocysts per treatment were transferred to each contralateral uterine horn such that each mother housed blastocysts from both control and treatment to control for the effect of mother on pregnancy outcome. The number of implantation sites and fetuses along with the fetal and placental weight and dimensions were recorded. For offspring studies, 5 morphological expanded or hatching blastocysts from a single treatment were transferred into each uterine horn of pseudo-pregnant Swiss female. Females were monitored for signs of pregnancy and on day 18 were individually housed and monitored for birth. All pups were given a paw pad dot tattoo on day 5 (i.e. 1 front left (1FL), 1 front right (1FR))to enable individual pups to be tracked and weighed on days 5, 7, 10, 14, 21 for measures of post natal growth. At day 21 post birth offspring were sexed and weaned from their mothers, grouped housed, maintained on standard chow and weighed weekly until 14 weeks of age

### Metabolic and body composition assessment of offspring

Dual-energy X-ray absorptiometry (DEXA) analysis and glucose tolerance tests (GTT) of offspring were carried out at 4, 8 and 12 weeks of age. GTT was performed after 6 h of fasting by intra-peritoneal (IP) injection of 2 g/kg of 25% D-glucose solution. Tail blood glucose concentrations were measured using a glucometer (Hemocue, Angelholm, Sweden) at time points 0 (pre-bolus basal), 15, 30, 60 and 120 min. Data were expressed as mean blood glucose concentration per group as area under curve (AUC) for GTT.

DEXA was performed in a fasted state and animals were anaesthetized using 2% Avertin (0.015 ml/g body weight). A LUNAR PIXImus Densitometer with PIXImus2 2.10 software was used to record x-ray images and measurements of bone density, bone mass, lean mass and fat mass were derived.

### Blood metabolites analysis

Serum was collected after overnight fasting via cardiac puncture while mice were anaesthetized with Isoflurane (2%; Veterinary Companies of Australia). Whole blood was allowed to clot at 4°C for 10 min prior to serum collection after spinning at 4000 rpm for 10 min. Serum was stored at −80°C until analysis of glucose, cholesterol, nonessential fatty acids (FFA), triglycerides, and high-density lipoprotein (HDL) using a mulit-analyte analyzer (Cobas Integra 400, Roche Diagnostics). Serum insulin was measured by ELISA (Crystal Chem, United States). Organs and adipose depots (omental, dorsal, perirenal, retroperirenal, gondal) were dissected and weighed post mortem (at 17 weeks of age).

### Statistics

All data are presented as mean values ± SEM where appropriate. Levels of significance were set to P≤0.05 for all analyses. All statistical analysis was performed in SPSS (SPSS Version 18, SPSS Inc., Chicago, USA). Chi-square tests were performed on embryo development data. For offspring measures all data was first checked for normality using a Kolmogorov-Smirnov test and equal variance using a Levene's test. Data was analysed using a linear mixed model with a Sidak post hoc test, litter size was added as a fixed effect with mother ID and father ID as a random factor where appropriate. Glucose and insulin tolerance data was analysed by repeated measures ANOVA, with litter size fitted as a fixed factor.

## Results

### Effect of incubation with H_2_O_2_ on sperm function

Incubation for 1 h with 1500 µM H_2_O_2_ did not affect sperm progressive motility compared to control (45.3%±2.4 control; 41.5%±2.6 1500 µM H_2_O_2_, P>0.05) or sperm binding as assessed by the mean number of sperm bound to an oocyte (34.5±6.7 control; 36.5±7.4 1500 µM H_2_O_2_, P>0.05). However, there was a 12% increase in intracellular ROS levels in sperm after 1 h incubation with 1500 µM H_2_O_2_ (control 120.0±2.5 fluorescent units; 1500 µM H_2_O_2_ 134.4±3.7 fluorescent units, P<0.01). Sperm that was treated with 1500 µM H_2_O_2_ for 1 h also had significantly higher levels of MSR positive sperm (2.3 fold change, P<0.01) and also an increase in sperm positive for lipid peroxidation as measured by sperm positive for 4-HNE (1.3 fold change, P<0.05).

### The effect of sperm treatment with H_2_O_2_ on fertilization and embryo development

There was no effect of H_2_O_2_ on fertilization rates during IVF as assessed by 2-cell rates after 24 h of culture (61% control; 64% 1500 µM H_2_O_2_, P>0.05). By 48 h of culture there were a significant reduction in the numbers of embryos at the 4 to 8-cell stage in embryos created by sperm treated with H_2_O_2_ compared with controls (P<0.05, Table 1), with an increased numbers of arrested/delayed embryos (<4-cell stage). This delay remained evident at 80 h of culture with significantly fewer embryos developing to the blastocyst stage when the sperm was treated with H_2_O_2_ (P<0.05, Table 1) and continued to be observed at 96 h of culture (P<0.05, Table 1).

**Table 1:** Development of embryos derived from sperm treated with 1500 µM H_2_O_2_.

Treatment	8-cell 48 h of culture	Blastocyst Development 80 h of culture	Blastocyst Development 96 h of culture
Control	88.6%	47.9%	62.7%
H_2_O_2_	81.6%*	43.9%*	57.6%*

All percentages are expressed as a proportion of 2-cell cleavage. Control n = 1129 embryos, H_2_O_2_ n = 1152 embryos.

*Indicates significantly different from control (P<0.05).

### The effect of sperm treatment with H_2_O_2_ on blastocyst cell number and differentiation

**Figure 1:**
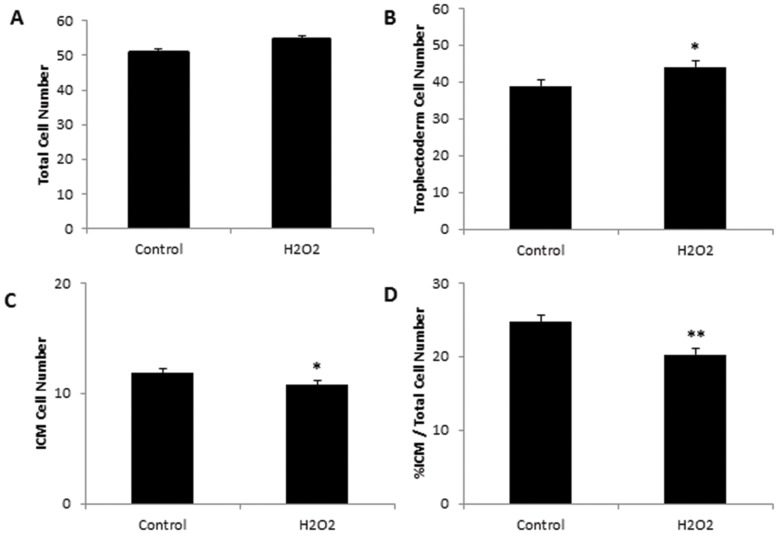
Effect of treating sperm with 1500 µM H_2_O_2_ on subsequent (A) blastocyst cell number, (B) trophectoderm cell number, (C) inner cell mass (ICM) cell number, (D) percentage (%) of ICM/total cell number. Control (n = 84 embryos), H_2_O_2_ (n = 79 embryos). Values are mean ± SEM.^*^ Indicates significantly different from control (P<0.05) ^**^ indicates significantly different from control (P<0.01).

There was no significant difference in total cell numbers of blastocysts from either treatment (P>0.05, Figure 1A). However, cell allocation was altered, with increased numbers of TE cells (P<0.05, Figure 1B) and decreased numbers of ICM (P<0.05, Figure 1C) in blastocysts produced by sperm treated with H_2_O_2_ compared with controls. This indicates that although there was no effect of sperm treatment with H_2_O_2_ on mitosis, cell differentiation was altered as evidenced by a significant reduction in the ICM:blastocyst cell number ratio (P<0.01, Figure 1D).

### The effect of sperm treatment with H_2_O_2_ on implantation and placental/fetal development

Blastocysts resulting from sperm treated with H_2_O_2_ tended to have lower implantation rates (P = 0.06) compared to controls although there was no significant difference in percentage of fetuses that developed (Table 2). Although there was also no statistically significant effect on placental weight or fetal weight, the fetal:placental weight ratio was significantly reduced in blastocysts produced by H_2_O_2_ sperm (P<0.05, Table 2). Furthermore day 18 fetuses sired by sperm treated with H_2_O_2_ had a significantly lower crown to rump length compared to fetuses resulting from control sperm (P<0.05, Table 2).

**Table 2:** Implantation and placental and fetal parameters at day 18 of gestation.

	Control	H_2_O_2_
**Implantation/blastocyst transferred (%)**	88.1	72.9+
**Fetal development/blastocyst transferred (%)**	47.6	39.6
**Fetal development/implantation (%)**	54.1	54.3
**Fetal weight (mg)**	1118.2±42.0	1059.4±56.9
**Placental weight (mg)**	147.3±5.9	167.9±11.1
**Fetal:placental ratio**	**7.7±0.3**	**6.46±0.5** *
**Crown-rump length (mm)**	**22.6±0.6**	**20.9±0.5** *

Values are mean ± SEM. n = 48 blastocyst stage embryos/treatment produced from 4 stud CBAF1 males transferred to contralateral uteri of 8 recipient females.

*indicates significantly different from control (also indicated by bold text) (P<0.05).

+ Indicates trending different from control P = 0.06. Survival rates post warming was between 90–95% for all treatment groups and did not differ statistically between treatment groups.

### The effect of sperm treatment with H_2_O_2_ on female offspring health

#### Body composition

**Figure 2:**
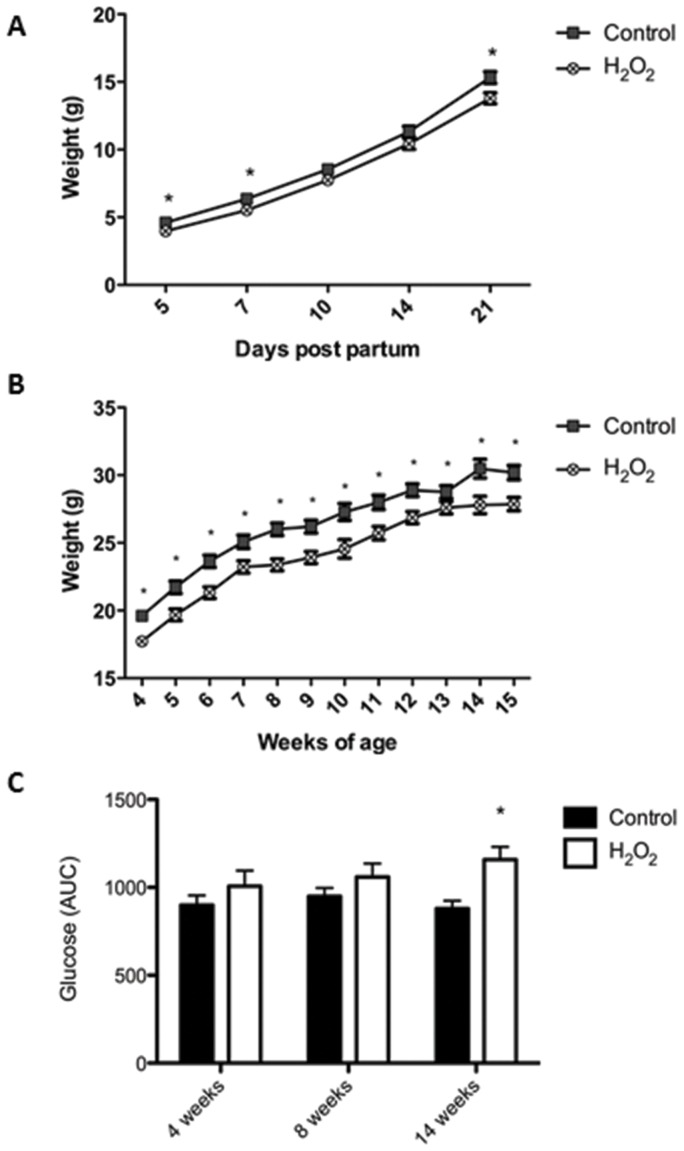
Effect of treatment of sperm with 1500 µM H_2_O_2_ on female offspring weight gain and glucose tolerance. (A) pre-weaning weight, (B) post weaning weight, (C) glucose tolerance (AUC) Control female (n = 7 from 6 litters), H_2_O_2_ female (n = 9 from 5 litters) representative of 15 stud CBAF1 males. Values are mean ± SEM.^*^ Indicates significantly different from control (P<0.05).

Female offspring sired from sperm treated with H_2_O_2_ were significantly lighter at postnatal day 5 and remained smaller throughout life (P<0.05, Figure 2A and 2B). At 4 weeks of age both groups had a similar fat mass as a percentage of body weight measured by DEXA (Table S1). By 14 weeks of age, females from H_2_O_2_ treated sperm had increased fat mass as a percentage of bodyweight compared to controls (Table S1). This increase in fat mass also coincided with a significant decline in lean mass at 14 weeks of age (P<0.05, Table S1). Furthermore, at 17 weeks of age upon post-mortem dissection female offspring sired by H_2_O_2_ treated sperm had significantly more adipose depots including omental fat, dorsal fat and gonadal fat which did not normalise when expressed as a percentage of body weight (P<0.05, Table 3). Interestingly, these females from H_2_O_2_ treated sperm also had smaller kidneys compared to control animals in terms of both absolute weight and as a proportion of total body weight.

**Table 3:** Post mortem female offspring body composition and fasting blood metabolites sired by control sperm or sperm treated with 1500 µM H_2_O_2_ at 17 weeks of age.

Tissue	Control	H_2_O_2_	Control (% body weight)	H_2_O_2_ (% body weight)
**Perirenal fat (g)**	0.024±0.01	0.037±0.01	0.090±0.03	0.131±0.02
**Retro peritoneal fat(g)**	0.033±0.02	0.074±0.02	0.126±0.07	0.262±0.05
**Omental fat (g)**	**0.177±0.04**	**0.295±0.04** *	**0.676±0.15**	**1.068±0.12** *
**Dorsal fat (g)**	**0.098±0.02**	**0.144±0.01** *	**0.370±0.05**	**0.533±0.04** *
**Gonadal fat (g)**	**0.22±0.13**	**0.56±0.10** *	**0.82±0.40**	**1.98±0.32** *
**Vastus lateralis (g)**	0.152±0.01	0.151±0.01	0.580±0.03	0.559±0.02
**Sum of fat depots (g)**	**0.55±0.21**	**1.11±0.17** *	**2.08±0.68**	**3.98±0.55** *
**Soleus (g)**	0.01±0.00	0.01±0.00	0.031±0.001	0.029±0.001
**Liver (g)**	1.10±0.05	1.08±0.04	4.17±0.13	4.02±0.11
**Pancreas (g)**	0.17±0.01	0.17±0.00	0.66±0.04	0.65±0.03
**Kidney (g)**	**0.19±0.01**	**0.17±0.01** *	**0.74±0.02**	**0.61±0.02** *
**Ovary (g)**	0.02±0.01	0.02±0.01	0.06±0.01	0.05±0.01
**Uterus (g)**	0.125±0.02	0.14±0.02	0.48±0.09	0.52±0.07
**Cholesterol (mM)**	2.99±0.24	3.04±0.22		
**Glucose (mM)**	**9.47±0.69**	**11.93±0.61** *		
**HDLs (mM)**	2.85±0.30	2.78±0.26		
**FFA (mM)**	1.01±0.20	1.08±0.15		
**Triglycerides (mM)**	0.66±0.10	0.69±0.09		
**Insulin (ng/ml)**	**0.06±0.01**	**0.09±0.01** *		

Values represent mean ± SEM. Control n = 7 females from 6 litters, H_2_O_2_ n = 9 females from 5 litters representative of 15 stud CBAF1 males.

*Indicates significantly different from control female offspring (also indicated by bold text) (P<0.05).

#### Metabolic health

Females sired by sperm treated with H_2_O_2_ had reduced glucose tolerance compared to control females that worsened as they aged such that there was an increase in the AUC at 14 weeks (Figure 2C, P<0.05). These females appeared to have a prolonged liver response with a delayed peak of blood glucose concentration at 8 weeks (15 mins control; 30 mins H_2_O_2_) and had increased fasting blood glucose concentration at all ages tested throughout life, including at 17-week post-mortem (P<0.05, Table 3). Furthermore, females sired from sperm treated with H_2_O_2_ also had elevated fasted blood insulin concentration (P<0.05, Table 3) while all other blood metabolites measured were not different (P>0.05, Table 3).

### The effect of sperm treatment with H_2_O_2_ on male offspring health

#### Body composition

On postnatal day 5 male offspring sired by sperm treated with H_2_O_2_ were significantly smaller however by postnatal day 7 this effect had dissipated and there was no difference in body weights to 14 weeks of age. Males sired by sperm treated with H_2_O_2_ had significantly higher percentage of body fat at 4 weeks of age (Table S2, P<0.05), which was normalised by 8 weeks of age, such that there were no differences in fat mass at 14 weeks of age (Table S2, P>0.05). Furthermore, at post mortem at 17 weeks the only difference in body weight was significantly smaller livers in male offspring sired by sperm treated with H_2_O_2._

#### Metabolic health

**Figure 3:**
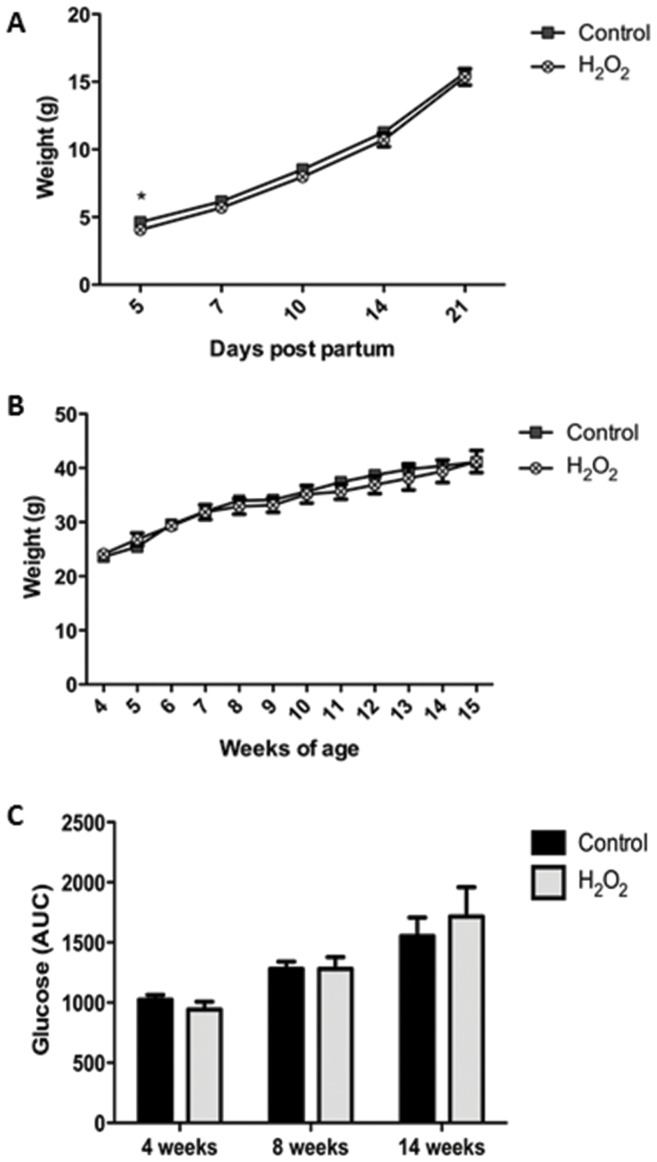
Effect of treatment of sperm with 1500 µM H_2_O_2_ on male offspring weight gain and glucose tolerance. (A) pre-weaning weight, (B) post weaning weight, (C) glucose tolerance (AUC). Control male (n = 14 mice from 6 litters), H_2_O_2_ male (n = 7 mice from 5 litters) representative of 15 stud CBAF1 males. Values are mean ± SEM.^*^ Indicates significantly different from control (P<0.05).

There was no change to glucose tolerance or fasting blood glucose concentrations nor were there any differences in blood insulin concentrations in male offspring sired by sperm treated with H_2_O_2_ (Figure 3, Table 4, p>0.05). Interestingly concentrations of FFAs and triglycerides were reduced in male offspring from H_2_O_2_, treated sperm (P<0.05, Table 4).

**Table 4:** Post mortem male offspring body composition and fasting blood metabolites sired by control sperm or sperm treated with 1500 µM H_2_O_2_ at 17 weeks of age.

Tissue	Control	H_2_O_2_	Control (% body weight)	H_2_O_2_ (% body weight)
**Perirenal fat (g)**	0.103±0.01	0.087±0.02	0.262±0.03	0.234±0.04
**Retroperitoneal fat (g)**	0.211±0.03	0.183±0.05	0.537±0.07	0.479±0.11
**Omental fat (G)**	0.469±0.04	0.447±0.07	1.203±0.09	1.194±0.15
**Dorsal fat (g)**	0.215±0.02	0.176±0.03	0.562±0.05	0.472±0.08
**Sum of fat depots(g)**	1.877±0.19	1.643±0.32	4.822±0.45	4.352±0.74
**Vastus lateralis (g)**	0.227±0.01	0.221±0.01	0.605±0.02	0.608±0.03
**Soleus (g)**	0.01±0.00	0.01±0.00	0.027±0.00	0.027±0.00
**Liver (g)**	**1.63±0.07**	**1.294±0.12** *	**4.32±0.13**	**3.55±0.21** *
**Pancreas (g)**	0.201±0.01	0.194±0.02	0.534±0.02	0.535±0.04
**Kidney (g)**	0.284±0.01	0.3±0.02	0.753±0.02	0.825±0.04
**Testis (g)**	0.108±0.01	0.103±0.01	0.288±0.02	0.281±0.03
**Gonadal fat (g)**	0.88±0.11	0.749±0.18	2.257±0.26	1.974±0.43
**Seminal vesicles (g)**	0.294±0.01	0.311±0.02	0.769±0.03	0.854±0.05
**Cholesterol (mM)**	4.28±0.25	4.39±0.35		
**Glucose (mM)**	9.96±0.74	10.31±1.04		
**HDLs (mM)**	3.97±0.21	4.23±0.29		
**FFA (mM)**	**1.55±0.15**	**0.85±0.21** *		
**Triglycerides (mM)**	**1.21±0.11**	**0.78±0.15** *		
**Insulin (ng/ml)**	0.11±0.01	0.11±0.01		

Values represent mean ± SEM Control n = 14 males from 6 litters, H_2_O_2_ n = 7 males from 5 litters representative of 15 stud CBAF1 males.

*Indicates significantly different from control male offspring (also indicated by bold text) (P<0.05).

## Discussion

It is now established that paternal health at the time of conception can impact the health of both the resultant pregnancy and offspring. A common observation in human paternal pathologies is increased levels of ROS in sperm and/or seminal fluid [30], implicating ROS as a potential causative factor in the transmission of paternal health cues to the offspring. Therefore this study established the direct impact of a pharmacologically induced increase in ROS in sperm. We determined that treatment of sperm with H_2_O_2_ resulted in poorer embryo development, reduced fetal growth, with the most pronounced effects observed in female offspring manifesting as altered body composition and glucose regulation.

Incubation of sperm with 1500 µM H_2_O_2_ did not affect sperm motility, binding or fertilisation, consistent with previous studies [31], [32]. However, as expected we did see increased concentrations of ROS in the sperm and evidence of oxidative damage in the form of increases in sperm mitochondrial ROS and lipid peroxidation. Analysis of the embryos that were generated with H_2_O_2_ treated sperm showed that embryo development was slowed at the 8-cell stage and blastocyst development was also delayed after both 80 hours and 96 hours of development. Analysis of blastocyst differentiation revealed no difference in mitosis however there appeared to be a skewing of differentiation favouring trophectoderm cells fate and a concomitant reduction in ICM cells compared to the control embryos. This impairment to blastocyst development mimics rodent models of paternal obesity whereby a delay in first/second cleavage events, compaction and reduced development to the blastocyst stage has been reported [8]. Furthermore, paternal obesity has been associated with reductions in blastocyst ICM, similar to the phenotype observed in this study [9], [33], [34]. The impact of altered preimplantation cell lineages can have significant long-term effects for the embryo as well as a whole organism perspective in relation to inappropriate stem cell allocation and numbers required for normal growth [35]. In addition studies have shown that decreased ICM cell number may be a causative factor for fetal growth retardation consistent with observations made in this study [36].

A reduction in the number of ICM cells in the blastocyst is reflective of reduced embryo viability post transfer [37]. Thus we transferred morphological similar blastocysts to assess implantation and fetal development rates. Implantation rates were slightly reduced for embryos created with H_2_O_2_ treated sperm although most striking was the reduction in fetal:placental weights, indicating a mismatch between the size of the placentas and the size of the fetuses. The fetuses also had significantly reduced crown-rump length on day 18, suggesting that placental insufficiency might have caused inadequate nutrient supply, resulting in a reduction of fetal size [38]. There are several models of placental insufficiency, usually induced at the later stages of pregnancy that result in smaller fetuses later in pregnancy and smaller offspring at birth [39]–[41]. These placental insult studies report smaller offspring, which are more susceptible to chronic diseases with an increased burden of metabolic disease in adulthood [42], [43].

Given the reduced fetal growth, we hypothesised that offspring metabolic health may have been similarly jeopardised by our model. Interestingly, we found that the most striking effects were observed in female offspring, which were smaller throughout their life. However they gained significantly more adipose tissue than the offspring from control untreated sperm and became glucose intolerant at 14 weeks of age. There is some recapitulation of the female offspring phenotype that was observed in other studies of paternal obesity, with reduced glucose tolerance and increased adiposity, implicating ROS as a mediator of the paternal transmission of obesity, at least in female offspring. In contrast, male offspring were only moderately affected early in life with increased adiposity at 4 weeks of age which dissipated as they aged and interestingly resulted in what appeared to be improved lipid metabolism at 14 weeks of age. Thus demonstrating sex-specific changes in the phenotype of offspring derived from H_2_O_2_ treated sperm which is also often seen in other models of offspring programming [11], [12], [35]. The direct mechanism for this sex specific effect is unknown however we do speculate that there is likely a difference in susceptibility to environmental perturbations between X and Y sperm which is evident in men exposed to exogenous environmental boron [44].

Through epidemiological studies the impact of low birth weight was initially correlated to an increased risk of non-communicable adult onset diseases such as stroke, high blood pressure, diabetes and heart disease [45], [46]. Building on the earlier studies the developmental origins of health and disease (DoHAD) hypothesis has emerged, stating that adaptations to environmental cues to which the gamete, embryo or developing fetus are exposed can lead to permanent alterations to internal programming that result in changes to offspring phenotype [45]. Growth characteristic and metabolic physiology are particularly sensitive, which can increase susceptibility to adult disease. Animal models demonstrate that offspring susceptibility to disease can be influenced by environmental factors surrounding gametes and embryos, such as maternal diet (low protein, undernutrition or vitamin intake) [35], [47], [48], maternal or paternal obesity [11],[49] and maternal/paternal exposure to endocrine disruptors or toxins [50]–[52]. Furthermore in vitro models such as suboptimal embryo culture or the addition of serum to culture media can affect offspring phenotype [53], [54]. All of the aforementioned exposures result in abnormal post-natal growth and increased risk of developing non-communicable disease including metabolic, cardiovascular and behavioural changes. A partial recapitulation of these offspring phenotypes have been observed in this study, namely increased adiposity and reduced glucose tolerance, albeit restricted to female offspring. It remains possible that the increased concentrations of ROS alters the genome/epigenome of sperm via a cascade of oxidative damage to DNA, RNA, proteins, mircoRNA and methyl side groups, warranting further investigations as potential downstream agents of paternal programming.

Mature sperm are particularly susceptible to ROS as the majority of their cytoplasm has been removed during spermiogenesis therefore their defence mechanisms are greatly diminished. While protamination compresses sperm DNA to a sixth of its original volume, protecting the DNA from environmental perturbations to a degree, some loci remain histone bound (∼1% of the mouse genome [55], [56]. DNA regions that remain histone bound are not only more susceptible to oxidative damage [57] through increased DNA damage and changes to sperm methylation patterns [20], [58] but these regions also harbour key genes important for embryo development [59]. These histone bound regions have been shown to be vital for paternal DNA replication following fertilization as well as activation of transcription of the paternal genome in early embryonic events [60]. Additionally paternal bound histone segments are not initially replaced by the oocyte and therefore any modifications to either these histones or the DNA within these regions are likely inherited in the embryo [61]. Therefore it has been hypothesied that oxidative stress to sperm targets these key histone bound regions, results in the transmission from sperm to embryo of permanent changes to the genome/epigenome of the developing embryo, altering the subsequent developmental profile [25], [56], [62]. The mechanism for this transmission into the early embryo during fertilization and cleavage remain to be elucidated however we speculate that that the oxidative damage to the sperm genome/epigenome (increased DNA adducts and alterations to methylation profiles) are transferred from the sperm into the embryo, which ultimately manifests as the observed offspring phenotype. The delay in embryo development seen might also result from the attempted repair of the damaged paternal genome/epigenome in the male pronuclei which results in a longer time to first and second cleavage events and subsequent blastocyst development via delays in paternal genome replication [63], [64]. This delay may then alter cell differentiation within the blastocyst and the development of implanted embryos, ultimately manifesting as altered post natal growth trajectories and non-communicable diseases in the adult offspring.

In conclusion this study has demonstrated that induction of oxidative stress in sperm using H_2_O_2_ caused oxidative damage to sperm, presumably to the sperm genome/epigenome, which subsequently reduced development rates of the preimplantation embryo and altered cell allocation in the blastocyst. This resulted in reduced embryo implantation, reduced fetal growth, increased adiposity, decreased lean mass and reduced glucose tolerance in female offspring. These data implicate ROS as one of the mechanisms responsible for the transmission of paternal health cues to offspring.

## Supporting Information

Table S1 Female offspring body composition assessed by DEXA at 4, 8 and 14 weeks of age.
(DOC)

Table S2 Male offspring body composition assessed by DEXA.
(DOC)
