# Supplementary material for: Oxidative Stress in Mouse Sperm Impairs Embryo Development, Fetal Growth and Alters Adiposity and Glucose Regulation in Female Offspring
Source: PLoS One. 2014 Jul 9;9(7):e100832. doi: 10.1371/journal.pone.0100832 (PMC4089912; doi:10.1371/journal.pone.0100832)
Supplement: Table S1 — Female offspring body composition assessed by DEXA at 4, 8 and 14 weeks of age. (DOC) [file pone.0100832.s001.doc]

**Table S1: Female offspring body composition assessed by DEXA at 4, 8 and 14 weeks of age**

|  | **4 weeks** | | **8 weeks** | | **14 weeks** | |
| --- | --- | --- | --- | --- | --- | --- |
|  | **Control** | **H_2_O_2_** | **Control** | **H_2_O_2_** | **Control** | **H_2_O_2_** |
| **Fat Mass (g)** | **1.78 ± 0.13** | **1.49 ± 0.21^*^** | 2.00 ± 0.16 | 1.61 ± 0.27 | 3.18 ± 0.53 | 4.00 ± 0.86 |
| **Fat mass (%)** | 9.14 ± 0.71 | 9.58 ± 0.53 | 8.70 ± 0.66 | 8.17 ± 1.07 | **11.9 ± 1.6** | **15.8 ± 2.7^*^** |
| **Bone Area (cm3)** | 6.53 ± 0.17 | 6.21 ± 0.15 | 8.28 ± 0.18 | 7.63 ± 0.29 | 9.20 ± 0.26 | 8.77 ± 0.42 |
| **Bone Mass (g)** | 0.27 ± 0.01 | 0.26 ± 0.05 | 0.44 ± 0.02 | 0.39 ± 0.03 | 0.55 ± 0.02 | 0.48 ± 0.03 |
| **Bone Mass (%)** | 1.43 ± 0.14 | 1.45 ± 0.13 | 1.89 ± 0.07 | 1.96 ± 0.12 | 2.06 ± 0.10 | 2.02 ± 0.17 |
| **Lean mass (g)** | 17.0 ± 0.4 | 15.9 ± 0.6 | **20.5 ± 0.5** | **17.9 ± 0.8^*^** | **22.8 ± 0.5** | **19.6 ± 0.9^*^** |
| **Lean mass (%)** | 89.4 ± 0.6 | 89.9 ± 0.5 | 89.4 ± 0.6 | 89.9 ± 1.03 | 86.0 ± 1.6 | 82.1 ± 2.6 |

Values represent mean ± SEM. Control n= 7 females H_2_O_2_ n=9 females.

* (and in bold text) significantly different from control offspring (P<0.05)
