# Supplementary material for: Oxidative Stress in Mouse Sperm Impairs Embryo Development, Fetal Growth and Alters Adiposity and Glucose Regulation in Female Offspring
Source: PLoS One. 2014 Jul 9;9(7):e100832. doi: 10.1371/journal.pone.0100832 (PMC4089912; doi:10.1371/journal.pone.0100832)
Supplement: Table S2 — Male offspring body composition assessed by DEXA. (DOC) [file pone.0100832.s002.doc]

**Table S2: Male offspring body composition assessed by DEXA**

|  | **4 weeks** | | **8 weeks** | | **14 weeks** | |
| --- | --- | --- | --- | --- | --- | --- |
|  | **Control** | **H_2_O_2_** | **Control** | **H_2_O_2_** | **Control** | **H_2_O_2_** |
| **Fat Mass (g)** | **1.84 ± 0.10** | **2.40 ± 0.15^*^** | 2.95 ±0.16 | 2.66 ± 0.24 | 6.17 ± 0.39 | 4.91± 0.58 |
| **Fat mass (%)** | **7.90 ± 0.40** | **9.90 ± 0.60^*^** | 9.96 ± 0.54 | 9.05 ± 0.82 | 16.60± 0.75 | 14.20± 1.10 |
| **Bone Area (cm3)** | 6.95 ± 0.16 | 6.62 ± 0.25 | 8.96 ± 0.19 | 8.87 ± 0.28 | 9.40 ± 0.16 | 9.59 ± 0.25 |
| **Bone Mass (g)** | 0.30 ± 0.01 | 0.29 ± 0.02 | 0.50 ± 0.02 | 0.49 ± 0.02 | 0.56 ± 0.01 | 0.53 ± 0.02 |
| **Bone Mass (%)** | **1.31 ± 0.03** | **1.20 ± 0.05^*^** | 1.68 ± 0.04 | 1.68 ± 0.06 | 1.52 ± 0.04 | 1.55 ± 0.06 |
| **Lean mass (g)** | 21.1 ± 0.60 | 21.6 ± 0.97 | 26.48± 0.64 | 26.07± 0.97 | 29.96± 0.59 | 28.68 ± 0.87 |
| **Lean mass (%)** | **90.8 ± 0.38** | **88.9 ± 0.58^*^** | 88.37± 0.52 | 89.27± 0.78 | 81.85 ± 0.73 | 84.25 ± 1.08 |

Values represent mean ± SEM. Control n= 14 males H_2_O_2_ n=7 males.

* (and in bold text) significantly different from control offspring (P<0.05)
